# Exergy Analysis of a Convective Heat Pump Dryer Integrated with a Membrane Energy Recovery Ventilator

**DOI:** 10.3390/e27020197

**Published:** 2025-02-13

**Authors:** Anand Balaraman, Md Ashiqur Rahman, Davide Ziviani, David M. Warsinger

**Affiliations:** School of Mechanical Engineering, Purdue University, 585 Purdue Mall, West Lafayette, IN 47907, USA; anandgbea@gmail.com (A.B.); rahma144@purdue.edu (M.A.R.); dziviani@purdue.edu (D.Z.)

**Keywords:** membrane dehumidifier, drying, heat pump, vapor-selective membrane, dehumidification

## Abstract

To increase energy efficiency, heat pump dryers and membrane dryers have been proposed to replace conventional fossil fuel dryers. Both conventional and heat pump dryers require substantial energy for condensing and reheating, while “active” membrane systems require vacuum pumps that are insufficiently developed. Lower temperature dehumidification systems make efficient use of membrane energy recovery ventilators (MERVs) that do not need vacuum pumps, but their high heat losses and lack of vapor selectivity have prevented their use in industrial drying. In this work, we propose an insulating membrane energy recovery ventilator for moisture removal from drying exhaust air, thereby reducing sensible heat loss from the dehumidification process and reheating energy. The second law analysis of the proposed system is carried out and compared with a baseline convective heat pump dryer. Irreversibilities in each component under different ambient temperatures (5–35 °C) and relative humidity (5–95%) are identified. At an ambient temperature of 35 °C, the proposed system substantially reduces sensible heat loss (47–60%) in the dehumidification process, resulting in a large reduction in condenser load (45–50%) compared to the baseline system. The evaporator in the proposed system accounts for up to 59% less irreversibility than the baseline system. A maximum of 24.5% reduction in overall exergy input is also observed. The highest exergy efficiency of 10.2% is obtained at an ambient condition of 35 °C and 5% relative humidity, which is more than twice the efficiency of the baseline system under the same operating condition.

## 1. Introduction

### 1.1. Background

Drying is a process of moisture removal from materials that requires both heat and mass transfer, as water is evaporated and then removed. It is an energy-intensive process, accounting for 10–25% of U.S. industrial energy use and contributing to greenhouse gas emissions [1,2]. Moreover, clothes dryers that typically use electrical resistive heating account for 4% of the total US residential energy consumption [3]. Convective drying is the most popular method of drying due to its low capital and operating costs for applications such as food [4], agriculture [5], biological [6], pharmaceutical [7], and garment industries [8,9]. A convective dryer removes moisture from wet products and condenses excess moisture from the air stream. However, the convective drying process requires significant thermal energy, often generated by burning fossil fuels [10]. Different energy-saving strategies, like exhaust air recirculation and waste heat exchange, can be applied to create an efficient convective drying system [11,12]. The DOE’s (Department of Energy, USA) vision on industrial decarbonization identified the electrification of high-temperature processes as a potential key pillar to significantly reduce energy and carbon emissions [13]. Towards this, heat pump dryers are emerging as an alternative to the traditional fossil fuel-based convective drying systems. However, moisture removal through condensation dehumidification in heat pump dryers is energy-intensive and requires larger reheating energy. Thus, there is a huge potential to develop an energy-efficient moisture removal process for the heat pump dryer.

### 1.2. Convective Heat Pump Dryer and Dehumidification Techniques

A heat pump dryer (HPD) can be operated with open- and closed-loop configurations [13,14]. An open heat pump dryer pulls in ambient air and heats it to the desired temperature, and then hot and humid air leaves the system after drying. On the other hand, in a closed-loop heat pump dryer, the dryer’s exhaust warm and humid air is passed through the evaporator (a component of the vapor compression cycle) to remove the water vapor by condensation, then reheated in the condenser and recirculated to the dryer [15,16]. Tunckal et al. studied three different modes (closed, open, and partially open cycles) of heat pump drying operating at a drying temperature of 40 °C. The highest average coefficient of performance (COP) of 2.50 was observed in the closed system [17]. However, due to condensation dehumidification, heat pump processes become energy-intensive. Among the components, the evaporator [18] and compressor cause significant exergy destruction due to the large temperature difference between the evaporator and humid air, as well as the high compressor ratio [19]. As a result, various configurations and hybrid systems have been developed to enhance the dehumidification process and improve HPD performance.

Desiccant-based drying is an alternate method that uses hygroscopic materials (desiccants) to absorb/adsorb water molecules from the air stream while releasing heat during dehumidification, lowering the sensible heat required for drying. However, the desiccant material needs additional heating for regeneration before it can be used in the next drying cycle [20,21]. Rane et al. found that a liquid-based desiccant drying system resulted in a 227% increase in SMER (specific moisture extraction rate) and a 56% increase in energy savings compared to a biomass-fueled conventional hot air-drying system [20]. A heat pump dryer coupled with a liquid desiccant dehumidifier can save between 30% and 60% of energy compared to a condensation dehumidification-based heat pump dryer [22]. Furthermore, solar thermal collectors can provide heat energy for the regeneration of the liquid desiccant, and the maximum SMER obtained from this hybrid process is 0.275 kg/kWh [23]. However, solar radiation energy is weather-dependent, and the capital cost is quite expensive. Due to the additional energy consumption of desiccant-based drying systems for regeneration, alternative methods like ultrasonic [24] and electro-osmosis [21] regeneration have been explored. Electro-osmosis is a recently emerging technique that uses an applied electric field on a desiccant to transport the polar water molecules toward the electrodes and expel them [21]. Challenges related to increased pressure drop in solid desiccant systems and liquid desiccant carryover, along with high energy requirements, necessitate alternate solutions for drying with low energy usage and reduced capital costs [25,26].

Membrane-based dehumidification is one of the emerging alternatives to condensation/desiccant dehumidification techniques [27,28]. The air-to-air MERV is widely used for dehumidification and sensible energy recovery in HVAC applications [28,29,30,31]. It has a vapor-selective membrane and drives water vapor from the feed to permeate stream using the partial pressure difference across the membrane. Vacuum pumps can increase the transmembrane pressure gradient, but they have limitations due to their high energy consumption [32]. However, a passive MERV is simpler in operation, compact, has no moving parts, low cost, and consumes less energy than the active vacuum-based membrane dehumidifier [33]. The average COP_eq_ of an MERV is 10 times greater than a single-stage vacuum membrane dehumidifier [34]. The latent effectiveness of an MERV is reported to increase by 7% for every 10% rise in the relative humidity of the feed stream, making it more advantageous in humid climatic zones [35]. Annual HVAC energy savings can reach up to 18–49% [35,36] and total exergy destruction decreases by more than 50% compared to when no MERV is used [37]. Consequently, overall exergy efficiency was reported to increase by 5–47% [37,38]. Further innovations on membrane materials and coatings have also been investigated to tailor their permeance, selectivity, thermal conductivity, and sensible/latent effectiveness to specific applications [38]. Various membrane materials, including paper, cellulose, and polymers, have been explored for MERVs. Conventionally, MERV designs are not selective for water over air and rely on using similar pressures to avoid air mass exchange. However, these systems can be less leaky and can tolerate pressure differences if selective polymer membranes are used, as these can have high water vapor-to-air selectivity (ranging from 10^3^ to 10^7^). These membranes typically consist of active and support layers [39]. The selectivity of polymer membranes can be further enhanced with various active coating materials, such as sulfonated polyether ether ketone (SPEEK), Pebax, polybenzimidazole (PBI), and NEXAR. The support layer, made from materials like polyvinylidene fluoride (PVDF), polyacrylonitrile (PAN), polyethersulfone (PES), and polysulfone (PSU), provides the necessary mechanical strength [34]. However, heat loss through these membranes is unavoidable, with average sensible and latent effectiveness typically ranging from 0.5 to 0.8 [39], and materials designed to reduce sensible heat transfer in MERV systems have rarely been explored. This work aims to provide insights into the potential of a near-isothermal dehumidification process and examine the impact of varying sensible and latent effectiveness in MERV systems when coupled with a heat pump dryer.

### 1.3. Literature Gap and Novelty

To date, only condensation dehumidification and desiccant dehumidification have been widely explored for moisture removal in the heat pump drying process. Both methods have limitations, either demanding high reheating energy for air in the drying process or desiccant regeneration energy. Membrane energy recovery ventilators are widely explored for building dehumidification to reduce cooling/heating coil size or energy demand and have shown significant energy savings compared to condensation/desiccant dehumidification. However, they have not yet been explored for high-temperature dehumidification applications such as industrial drying. Thus, we propose a passive MERV for moisture removal from the drying exhaust air in the convective heat pump drying process. Using an MERV removes latent load and minimizes sensible heat loss in the dehumidification process, thus greatly reducing reheating energy demand and improving overall performance. This also can reduce irreversibility associated with heat transfer due to high-temperature differences between the working fluid and dead state conditions. In this work, a second law analysis of the proposed system is carried out, comparing it with the baseline convective heat pump dryer to identify potential exergy destruction associated with each component under various operating conditions.

## 2. Methods

A detailed working principle and description of both the proposed and baseline systems are discussed in this section. The advantages of the proposed system over the baseline system are then analyzed and represented using a psychrometric chart and a T-s diagram. Further, details on modeling assumptions and methodology are discussed.

### 2.1. System Description

**Figure 1:**
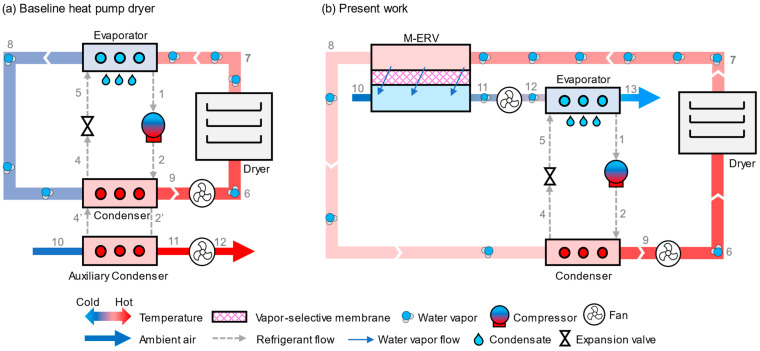
Schematic of (**a**) the baseline heat pump dryer, and (**b**) the proposed dryer with a membrane energy recovery ventilator. The baseline uses a heat pump to condense water vapor, where its hot condenser coil is placed to reheat the air stream, and then rejects excess heat to the ambient air through an auxiliary condenser. The proposed dryer removes water vapor with a membrane that exchanges vapor with ambient air, reducing heat loss and reheating energy. The heat pump that reheats the dehumidified air stream draws its heat from the humidified ambient air stream, thereby reducing the temperature rise.

A schematic of the baseline heat pump dryer and the present system are shown in Figure 1. In the baseline system, hot and dry air enters (6) the dryer, removes moisture from the drying material and enters the evaporator. Moisture is removed from the warm and humid air (7) through the cooling and dehumidification process in the evaporator. The obtained cold and dry air (8) is then reheated in the condenser to the desired dryer inlet temperature. Since the evaporator and condenser are used in a closed loop, excess heat is rejected in the auxiliary condenser by exchanging heat with the ambient air (10). However, in the present system, moisture is removed using a membrane dehumidifier. Unlike the baseline system, the membrane dehumidifier removes moisture using a partial pressure gradient between the feed (7) and permeate air stream/ambient air (10). Thus, heat loss in the membrane dehumidifier is much smaller, which greatly reduces the heat pump load and power consumption. Notably, the air parameters at state 10 are the same in both the baseline (Figure 1a) and the proposed system (Figure 1b). As shown in Figure 1b, the warm humid air from the dryer (7) enters the membrane dehumidifier; consequently, ambient air (10) enters the permeate side of the membrane dehumidifier. Due to the partial pressure difference, moisture diffuses through the membrane and is taken away by the ambient air stream. The resulting dry air (8) leaving the membrane dehumidifier is then reheated in the condenser to the desired drying temperature. Ambient air leaving the membrane dehumidifier (11) then enters the evaporator of the heat pump, where it is cooled and dehumidified until it provides the desired heat load to the condenser of the heat pump. Since the evaporator exchanges heat with the ambient air, the present system does not require an auxiliary condenser, enabling the evaporator to operate at a higher temperature than the baseline system under certain operating conditions and allowing the entire condenser load to be used for reheating the drying air stream.

**Figure 2:**
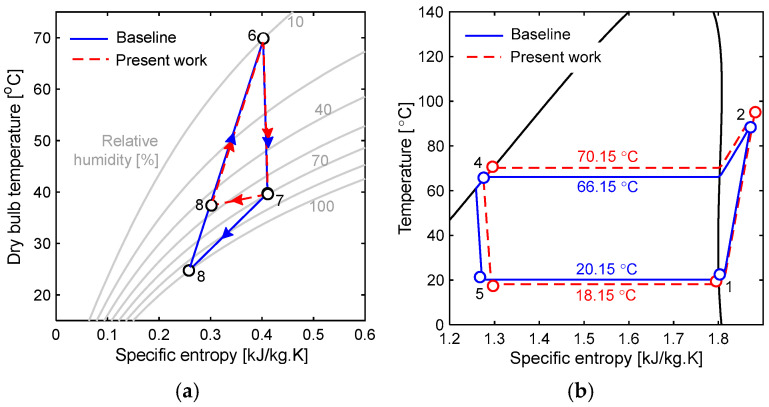
Representation of the baseline and proposed system in the T-s diagram (**a**) air-side T-s diagram, and (**b**) refrigerant-side T-s diagram.

The advantage of having a membrane dehumidifier to remove the moisture content from the drying air stream is depicted in the T-s diagram (Figure 2). As shown, the temperature loss in the dehumidification process (7–8) of the present system is 53% lower than that of the baseline system. Because of this, the temperature difference in air across the condenser is also 16% less than that of the baseline system. This results in a 37% smaller heat pump size for the present system. However, the evaporator operating temperature is 2 °C lower, and the refrigerant condensing temperature is 4 °C higher than the that of the baseline system. This may contribute to high energy consumption, but it is offset by energy savings due to a reduction in heat pump capacity.

### 2.2. Modeling Methodology

The modeling methodology of both the baseline and proposed system begins with determining the required heat pump size for a given dryer capacity and drying condition (i.e., drying temperature and relative humidity) at different ambient temperatures and relative humidity. A tray dryer with dimensions of 0.5 m × 0.5 m × 0.5 m is initially considered based on heuristics for sizing the dryer [28]. However, the total drying capacity will vary depending on the product density. In this study, a product with a density of 560 kg/m^3^ is used to estimate the total quantity of the materials (7 kg) to be dried in the tray dryer. Additional details on estimating product quantity for the specified dryer dimensions are presented in the following section. The following assumptions are considered for the modeling of both systems.

- The air inlet conditions at the dyer are 70 °C and 10% RH.
- The initial and final moisture content of the drying material is 90% w.b and 10% w.b, respectively.
- The tray thickness and distance between the tray are 0.02 m and 0.076 m, respectively [40].
- The air velocity for the thin-layer drying process is 1 m/s [41].
- The evaporator superheat and condenser subcooling in the heat pump unit are 5 °C, respectively [42,43].
- The pinch point temperature in the evaporator and condenser is 5 °C, respectively [44].
- Compressor isentropic efficiency is 70%, and overall fan efficiency is 70% [45].
- The sensible effectiveness of the membrane dehumidifier is initially assumed to be 0.7 [39] and is varied between 0.7 and 0.1 to assess the influence of insulative membranes.
- The latent effectiveness of the membrane dehumidifier is 0.7 [39].
- The air pressure drop across the heat exchanger is 250 Pa [46].
- The air pressure drop in the dryer and membrane dehumidifier is 100 Pa and 125 Pa, respectively [47,48].

Following the assumptions, the total quantity of the material that can be accommodated in the tray dryer is calculated following the procedure given by Alexander et al. [40]. Based on the assumed dimensions of the tray dryer, the number of trays the dryer can accommodate is calculated using the tray thickness and the distance between the trays. Since the bed thickness is assumed to be 0.01 m for the thin-layer drying [41], the maximum product volume (0.0025 m^3^) in each tray is calculated using the bed thickness (0.01 m) and the tray area (0.25 m^2^). The total product volume (0.0125 m^3^) is the product of the total number of trays (5) and the product volume (0.0025 m^3^) per tray. Finally, the total kilograms of material (7 kg) that the dryer can accommodate is the product of the material density and the total product volume. The air flow rate (0.25 kg/s) is calculated using the tray cross-sectional area (0.25 m^2^) and the air velocity in the dryer (1 m/s). Similarly, the required tray dimensions for a given quantity of the drying material can be estimated by reversing the calculations. However, different material densities will yield different dryer dimensions. Thus, a more practical approach is considered in this work, which quantifies the drying material for a given tray’s dimensions.

The dryer outlet air conditions are calculated using the following equation.

(1) $$T_{a,7}=T_{a,6}-\eta_{dryer}\left(T_{a,6}-T_{ideal}\right)$$

(2) $$\omega_{a,7}=\omega_{a,6}-\eta_{dryer}\left(\omega_{a,6}-\omega_{ideal}\right)$$

here, $\eta_{dryer}$ is dryer efficiency, while $T_{ideal}$ and $\omega_{ideal}$ denote the dryer outlet temperature and humidity ratio under the ideal operating condition, respectively, where drying occurs adiabatically, and the outlet air reaches 100% relative humidity [49].

In both of the systems, the dryer outlet air stream is dehumidified until it reaches the target humidity ratio, which is equivalent to the dryer inlet humidity ratio. Considering the baseline system, the evaporator heat load is initially estimated using an air-side energy balance. The target evaporator operating temperature is iterated by discretizing the air- and refrigerant-side heat transfer, assessing its temperature profile to meet the desired pinch condition. Thus, the required refrigerant flow rate is calculated as

(3) $${\dot{m}}_{ref,baseline}=\frac{{\dot{Q}}_{evap}}{h_{1}-h_{5}}=\frac{{\dot{m}}_{air}\left(h_{7}-h_{8}\right)}{h_{1}-h_{5}}$$

Similarly, the internal condenser heat load is calculated using an air-side energy balance. At a given compressor isentropic efficiency, the refrigerant outlet temperature at the compressor is calculated by iterating the condensing temperature until it meets the desired condenser heat load and pinch temperature assumption. The excess heat load is rejected in the external condenser where the required air flow rate is also iterated until it meets the pinch condition.

Considering the present system, moisture from the dryer outlet air stream is removed in the membrane dehumidifier using a partial pressure driving force across the membrane. The ambient air flow rate at the permeate side is iterated to remove the desired moisture content at given sensible (${ɛ}_{sensible}$) and latent effectiveness (${ɛ}_{latent}$) values of the membrane dehumidifier.

(4) $${ɛ}_{sensible}=\frac{{\left(\dot{m}c_{p}\right)}_{hot}\left(T_{a,7}-T_{a,8}\right)}{{\left(\dot{m}c_{p}\right)}_{min}\left(T_{a,7}-T_{a,10}\right)}=\frac{{\left(\dot{m}c_{p}\right)}_{cold}\left(T_{a,10}-T_{a,11}\right)}{{\left(\dot{m}c_{p}\right)}_{min}\left(T_{a,10}-T_{a,7}\right)}$$

(5) $${ɛ}_{latent}=\frac{{\left(\dot{m}c_{p-h}\right)}_{hot}\left(P_{v,7}-P_{v,8}\right)}{{\left(\dot{m}c_{p-h}\right)}_{min}\left(P_{v,7}-P_{v,10}\right)}=\frac{{\left(\dot{m}c_{p-h}\right)}_{cold}\left(P_{v,10}-P_{v,11}\right)}{{\left(\dot{m}c_{p-h}\right)}_{min}\left(P_{v,10}-P_{v,7}\right)}$$

Here, $c_{p-h}$ is the specific humidity capacity, defined as a ratio between the change in the humidity ratio (Δω) to the change in the vapor partial pressure (ΔP) in the membrane dehumidifier [50].

The following conditions given in Equations (6) and (7) are considered to check if the system removes the target moisture content without initiating condensation due to excess sensible heat loss in the dryer air stream or the oversaturation of ambient air. Condensation in the membrane dehumidifier is undesirable because it may block the air passage, which will lead to reduced effectiveness and damage it over time.

(6) $$\left(P_{v,8}=P_{v,6}\right)$$

(7) $$\left(P_{v,8}\leq P_{sat,8}\right),\;\left(P_{v,11}\leq P_{sat,11}\right)$$

The warm and humid ambient air leaving the membrane dehumidifier is cooled in the evaporator until it provides the desired heat load to the condenser. The condenser heat load can be calculated using an air-side energy balance across the condenser. The condenser and evaporator temperature are iterated until they meet the pinch assumption and the desired heat loads.

The total drying time ($t_{d}$) to remove the total moisture content is calculated as

(8) $$t_{d}=\frac{m_{w}}{{\dot{m}}_{air}\left(\omega_{7}-\omega_{6}\right)}$$

The total power consumption (${\dot{W}}_{total}$) is estimated as

(9) $${\dot{W}}_{comp}={\dot{m}}_{ref}\left(h_{r,1}-h_{r,2}\right)$$

(10) $${\dot{W}}_{fan}=\frac{{\dot{V}}_{air}∆P}{\eta_{fan}}$$

(11) $${\dot{W}}_{total}={\dot{W}}_{comp}+{\dot{W}}_{fan-1}+{\dot{W}}_{fan-2}$$

Here, $h_{r}$ is the refrigerant enthalpy, ${\dot{m}}_{ref}$ is the refrigerant mass flow rate, ${\dot{V}}_{air}$ is the volumetric flow rate of air, ∆P is the fan pressure drop, and $\eta_{fan}$ is fan efficiency, respectively. Further, ${\dot{W}}_{comp}$ is compressor work, ${\dot{W}}_{fan}$ is fan work, and ${\dot{W}}_{total}$ is the total work, respectively.

The exergy analysis is then carried out by estimating exergy destruction in each component. The impact of heat transfer that raises the temperature of the material is very small/negligible compared to the heat transfer that evaporates water and heat loss through the wall [51]. Considering that the dryer is well insulated, the drying process can be considered analogous to the adiabatic evaporative cooling process [49,52,53,54]. Hence, the exergy destruction in the dryer can be calculated as

(12) $${\dot{I}}_{dryer}={\dot{Ex}}_{6}-{\dot{Ex}}_{7}+{\dot{Ex}}_{evap}$$

Here, ${\dot{I}}_{dryer}$ is the exergy destruction of the dryer and $\dot{Ex}$ is the exergy transfer rate, which is the product of the mass flow rate ($\dot{m}$) and specific exergy $\left(e=\left(h-h_{o}\right)-T_{o}\left(s-s_{o}\right)\right)$ at the respective state points. The exergy transfer rate due to evaporation is calculated as

(13) $${\dot{Ex}}_{evap}=\left[1-\frac{T_{o}}{T_{w}}\right]{\dot{m}}_{wv}h_{fg}$$

where $T_{w}$ is the temperature of water content in the material, which is considered as an average of the air inlet temperature and its wet bulb temperature. Here, the air inlet temperature is the maximum temperature that the water content can attain, while the wet bulb temperature is the lowest possible temperature the air can attain due to evaporation, and thus the average of these is considered for the analysis.

Further details on the exergy balance of the heat pump components and membrane dehumidifier are given in Table 1. Based on the exergy destructions in each component, the total exergy destruction (${\dot{I}}_{t}$) in the baseline system and present system are estimated as follows

(14) $${\dot{I}}_{t,baseline}={\dot{I}}_{dryer}+{\dot{I}}_{evaporator}+{\dot{I}}_{cond-1}+{\dot{I}}_{cond-2}+{\dot{I}}_{comp}+{\dot{I}}_{exp}+{\dot{I}}_{fan-1}+{\dot{I}}_{fan-2}$$

(15) $${\dot{I}}_{t,present\;system}={\dot{I}}_{dryer}+{\dot{I}}_{md}+{\dot{I}}_{evaporator}+{\dot{I}}_{cond}+{\dot{I}}_{comp}+{\dot{I}}_{exp}+{\dot{I}}_{fan-1}+{\dot{I}}_{fan-2}$$

The exergy efficiency ($\eta_{e}$) of baseline and present system are defined as,

(16) $$\eta_{II,baseline}=\frac{{\dot{Ex}}_{in}-{\dot{I}}_{t,baseline}}{{\dot{E}x}_{in}}$$

(17) $$\eta_{II,present\;system}=\frac{{\dot{Ex}}_{in}-{\dot{I}}_{t,present\;system}}{{\dot{Ex}}_{in}}$$

where the ${\dot{E}x}_{in}$ is the total exergy input, which is the sum of all exergy input to the control volume of the system. The total exergy input includes the power consumption from the compressor, fans, and exergy input in water vapor entering the air stream in the drying process. Further, the state point properties of the air and refrigerant in the proposed system (at 20 °C and 95% of the ambient condition) are presented in Table 2.

## 3. Results and Discussion

In this section, the results of the exergy analysis of the baseline and present systems are presented and compared. The exergy analysis was conducted at different ambient temperatures (5 °C to 35 °C) and relative humidity levels (5% to 95%). The dead state temperature and relative humidity are assumed to match the ambient temperature and relative humidity, while the dead state pressure is set to the ambient pressure (1.013 bar). Initially, the exergy destruction in each component is presented and discussed. Then, the overall exergy efficiency of the systems is compared, and the influence of membrane sensible effectiveness on total exergy destruction and second law efficiency is presented. Further, exergy flow is visualized using a Grassman diagram.

### 3.1. Exergy Destruction in Dryer

The dryer operates at the same inlet (70 °C and 10% RH) and outlet conditions (39 °C and 69% RH) at a given dryer efficiency (85%) in both the systems, across all ambient conditions. Changes in exergy destruction in the dryer are solely due to changes in the dead state, which are identical for both the baseline and present systems. Since the dead state is assumed to be equal to the ambient conditions, exergy destruction is highest at the lowest ambient temperature (5 °C). The decrease in ambient temperature increases the temperature difference, leading to greater irreversibility. The highest exergy destruction rate in the dryer is 2.66 kW at an ambient temperature of 5 °C and 95% RH.

### 3.2. Exergy Destruction in Heat Pump Components

#### 3.2.1. Exergy Destruction in Evaporator

**Figure 3:**
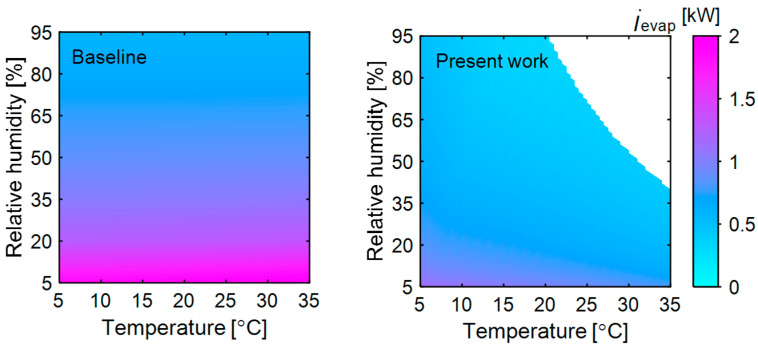
Exergy destruction in the evaporator of the heat pump in the baseline (**left**) and present system (**right**) as ambient temperature and relative humidity are varied. The membrane’s sensible and latent effectiveness (0.7 and 0.7, respectively), dryer air inlet conditions (70 °C and 10% RH), dryer capacity (7 kg), dryer air velocity (1 m/s), superheat (5 °C) and subcooling (5 °C) in the heat pump are kept constant as specified in the modeling assumptions in Section 2.2.

The exergy destruction in the evaporator of the baseline and present system is compared in Figure 3. In the baseline system, the evaporator operates at the same temperature, irrespective of the changes in ambient conditions. For instance, the evaporator operating temperature (20 °C), air inlet (39 °C and 69% RH) and outlet conditions (25 °C and 100% RH) at the evaporator remain constant for a given drying condition (70 °C and 10%) across all ambient temperatures and relative humidities. A nearly negligible change in the exergy destruction rate (<5%) was observed in the evaporator with changes in the dead state temperature at a given relative humidity. As the air undergoes the cooling and dehumidification process in the evaporator, its useful work relative to the reference dead state decreases. Meanwhile, as the refrigerant becomes saturated vapor, its useful work potential increases relative to the dead state. These changes in the specific exergies of the air and refrigerant offset each other, resulting in insignificant changes in the exergy destruction rate. However, significant changes (greater than 31%) were observed, with variations in dead state relative humidity at a given temperature. This occurs because dry air has a low dew point temperature, resulting in a large difference between the dead state and working fluids, which increases irreversibility. As the dead state relative humidity increases, irreversibility decreases, resulting in lower exergy destruction.

In contrast to the baseline, in the present system, the evaporator exchanges heat with ambient air leaving the membrane dehumidifier and cooling it until it provides the desired heat load to the condenser. The exergy destruction in the evaporator of the present system is shown in Figure 3. The contour region represents the operational boundary; outside this region, the system does not achieve the required moisture removal. At low ambient temperature (5 °C) and relative humidity (5%), the ambient air has low energy, becoming warm and humid (29 °C and 89% RH) after absorbing heat and moisture in the membrane dehumidifier. However, since the airflow rate from the membrane dehumidifier is relatively low, the evaporator needs to cool the air to a lower temperature (10 °C) to meet the condenser’s heat load requirement. This necessitates a lower evaporator operating temperature (5 °C). Thus, a significant temperature difference between the air and refrigerant results in high exergy destruction in the present system at low ambient temperature and relative humidity. Conversely, at high ambient temperature (35 °C) and relative humidity (40%), the air possesses high energy, and the resulting air from the membrane dehumidifier is also at a high temperature (38 °C). As a result, the evaporator cools the air only to 26 °C and can operate at 21 °C to deliver the required heat to the condenser. This smaller temperature difference between the dead state and working fluids leads to a lower exergy destruction rate (0.42 kW). This demonstrates the advantage of evaporator heat exchanges with the ambient air than with the drying air stream. 

#### 3.2.2. Exergy Destruction in Condenser

**Figure 4:**
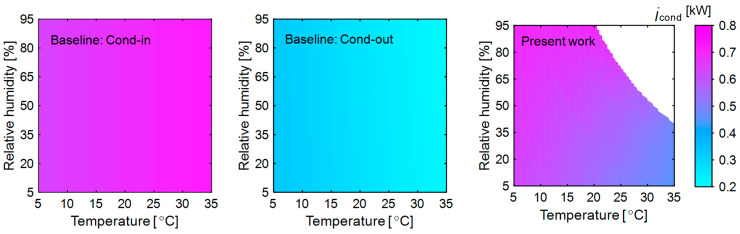
Exergy destruction in the condenser of the heat pump in the baseline and present systems at various ambient temperature and relative humidity. The other variables are kept constant as mentioned in Figure 3.

The exergy destruction in the condenser of the baseline and present system is shown in Figure 4. The baseline system includes the main condenser and an auxiliary condenser (Figure 1a). The main condenser exchanges heat with the cooled and dehumidified (25 °C and 100% RH) air from the evaporator. Since all the operating conditions remain constant across all ambient temperatures and no phase change occurs on the air side, the changes in the exergy destruction rate are insignificant. The auxiliary condenser exchanges heat with the ambient air. A high air flow rate is required at high ambient temperatures (35 °C) to remove the same heat load (3.85 kW). Consequently, less temperature is gained across the condenser (52% lesser than at 5 °C of ambient temperature) and the reduced temperature difference with the dead state results in lower exergy destruction.

In the present system, at low ambient temperature (5 °C), heat loss in the membrane dehumidifier is significantly higher (85%) than at a high ambient temperature (35 °C). Additionally, when the ambient temperature is held constant at 5 °C, higher relative humidity (95%) requires a greater air flow rate compared to low relative humidity (5%), leading to a 19% increase in heat loss. Therefore, the temperature difference between the air entering the condenser and condensing temperature is high. Along with this, the large temperature difference between the dead state and air entering the condenser contributes to high exergy loss. As the ambient temperature increases, both heat loss and temperature difference decrease, reducing exergy destruction.

#### 3.2.3. Exergy Destruction in Compressor

**Figure 5:**
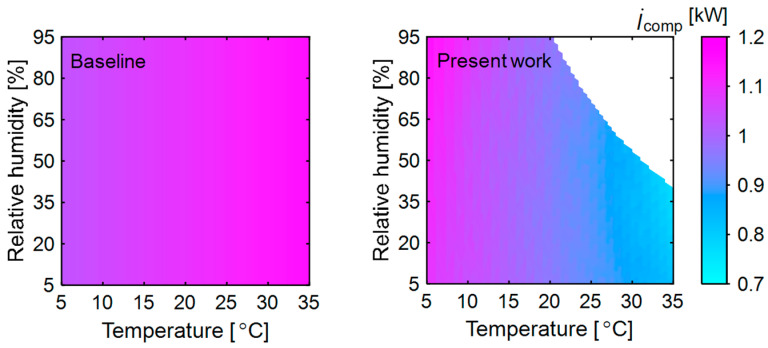
Exergy destruction in the compressor of heat pump in the baseline and present system at different ambient temperatures and relative humidities. The other variables are kept constant as mentioned in Figure 3.

The exergy destruction rate in the compressor for both the baseline and present system is shown in Figure 5. In the baseline system, the refrigerant flow rate and the temperatures of the refrigerant entering and leaving the compressor remain constant across all ambient conditions. So, entropy generation is steady, with exergy destruction increasing only as the dead state temperature rises. In the present system, as discussed, significant heat loss occurs in the membrane dehumidifier, particularly at low ambient temperatures. Additionally, higher ambient relative humidity requires a larger air flow rate to achieve the desired moisture removal, increasing heat loss and reheating energy demand. At a low ambient temperature (5 °C), the temperature lift in the heat pump is 63 °C, compared to 55 °C at a high ambient temperature (35 °C). This greater temperature lift, coupled with a higher refrigerant flow rate, results in high entropy generation at low ambient temperatures. However, the temperature lift and its impact on exergy destruction may vary depending on specific operating conditions.

Compared to the baseline system, exergy destruction in the present system is significantly lower at elevated ambient temperatures. Drying requires the addition of heat at high temperatures in the condenser. At high ambient temperatures, heat loss in the membrane dehumidifier is less, and warmer air enters the condenser compared to the operation at low ambient temperatures. As a result, the smaller temperature difference across the condenser requires a lower condenser heat load, leading to reduced exergy destruction. Additionally, the smaller temperature difference between the air at the condenser and the dead state at high ambient temperatures further lowers exergy destruction. In contrast, the temperature lift in the baseline system remains constant at all ambient temperatures. In the present system, irreversibility at low ambient temperature (<10 °C) is greater than that in the baseline system due to the low evaporator operating temperature and high temperature lift.

### 3.3. Exergy Destruction in Membrane Dehumidifier

**Figure 6:**
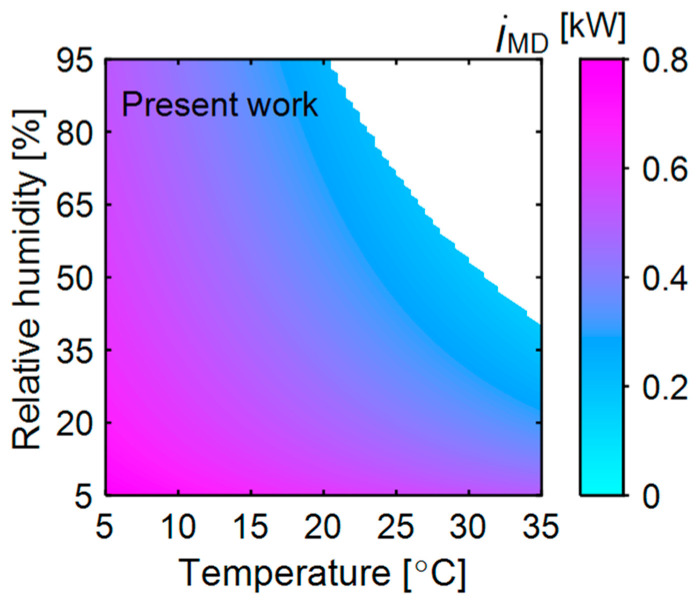
Exergy destruction in the membrane dehumidifier in the present system at various ambient temperatures and relative humidities. The other variables are kept constant as mentioned in Figure 3.

Exergy destruction in the membrane dehumidifier is shown in Figure 6. At low ambient temperature (5 °C) and relative humidity (5%), high temperature and the partial pressure difference between the feed inlet (39 °C and 69%, respectively) and the dead state contribute to high exergy loss. Thus, the exergy destruction decreases as the ambient temperature and relative humidity increase and reaches the lowest value (0.17 kW) at the ambient condition of 35 °C and 40% RH.

### 3.4. Exergy Efficiency

**Figure 7:**
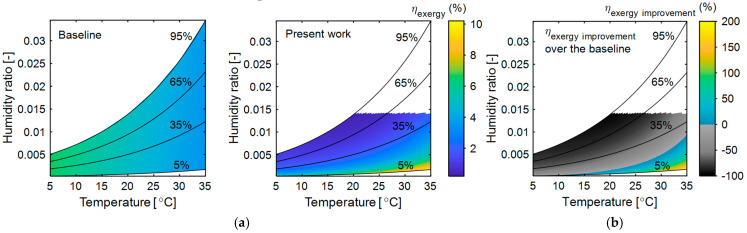
(**a**) Exergy efficiency of the baseline and present system at various ambient temperatures and relative humidity levels. The other variables are kept constant, as mentioned in Figure 3. (**b**) The exergy efficiency improvement of the proposed system over the baseline system.

Figure 7 shows the exergy efficiency of the baseline and present system at different ambient temperatures and relative humidity levels. In the baseline system, heat pump components such as the evaporator, compressor, internal condenser, and expansion device contribute minimally to exergy destruction at low ambient temperatures (5 °C). This minimal exergy destruction of each component synergistically results in high exergy efficiency at low ambient temperatures. Thus, the heat pump components have a greater influence on the baseline system’s exergy efficiency than the dryer. In contrast, in the present system, at a high ambient temperature (>25 °C), the temperature difference between the dead state and the working fluids is much smaller than in the baseline system. Additionally, exergy destruction in the membrane dehumidifier and dryer is also less at high ambient temperatures. This, combined with reduced exergy input, results in high exergy efficiency (10%) at high temperatures (35 °C and 5% RH). However, as relative humidity increases at a given ambient temperature, the temperature loss in the membrane dehumidifier increases, requiring larger exergy input and causing higher exergy destruction. Consequently, exergy efficiency is less (2.26%) at high relative humidity (40%) at a given ambient temperature (35 °C).

At low ambient temperatures, the total exergy input in the present system is very similar to that of the baseline system, with only a 1% difference. The high temperature loss in the membrane dehumidifier causes minimal net energy savings, and the evaporator operates at a lower temperature (<15 °C) compared to the baseline system. This results in high exergy destruction due to the lower evaporator operating temperature, along with additional exergy destruction in the membrane dehumidifier, leading to lower exergy efficiency. However, the present system’s exergy efficiency is much higher than the baseline system at high ambient temperature (>20 °C) and low relative humidity (<30%). The proposed system performs better under ambient conditions that minimize temperature loss in the membrane dehumidifier and that enable a higher evaporator operating temperature. The overall improvement in the exergy efficiency of the proposed system compared to the baseline system under different ambient conditions is shown in Figure 7 (right). Although the exergy efficiency of the present system appears to be better than that of the baseline system only within a smaller operating range, the heat pump capacity of the present system is reduced by up to 50% compared to the baseline system for drying the same quantity of material. This subsequently contributes to reduced energy consumption and exergy input.

**Figure 8:**
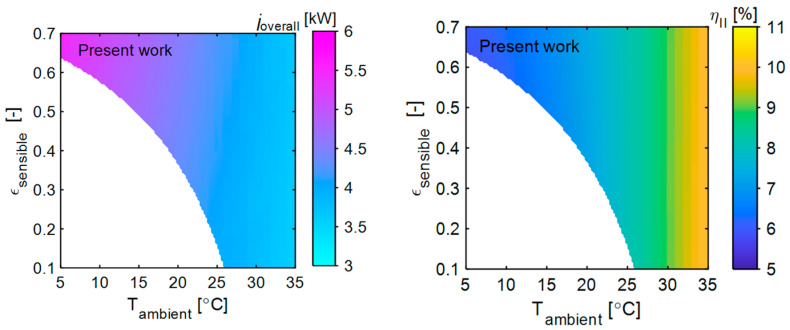
The influence of the membrane’s sensible effectiveness on the overall exergy destruction of the present system at different ambient temperatures (**left**). The influence of the membrane’s sensible effectiveness on the second law efficiency of the present system at different ambient temperatures (**right**). In both cases, ambient relative humidity and membrane latent effectiveness were kept constant at 5% and 0.7, respectively. The non-contour region represents that the present system cannot remove the desired moisture content and it can be considered the non-operating zone.

The exergy analysis so far has considered the membrane dehumidifier’s sensible and latent effectiveness as 0.7 each, respectively [40]. However, our recent studies explored novel nano-coatings for thermally insulative, highly water vapor-selective, and permeable membranes designed for high-temperature dehumidification applications. To assess the impact of a thermally insulative vapor-selective membrane on the exergy performance of the present system, the sensible effectiveness of the membrane dehumidifier varied between 0.1 and 0.7. The overall exergy destruction and exergy efficiency of the present system at different sensible effectiveness across various ambient temperatures are studied and presented in Figure 8.

As shown, with a constant sensible effectiveness of 0.7, the overall exergy destruction (5.55 kW) is high at a low ambient temperature (5 °C) and gradually decreases as the ambient temperature increases (to 3.64 kW at 35 °C). At high ambient temperatures, the temperature difference between the dead state and the working fluids is smaller, reducing exergy destruction. At a constant ambient temperature, the overall exergy destruction increases as the sensible effectiveness of the membrane dehumidifier rises. However, this alone does not fully explain the system’s behavior, as exergy input also increases with higher sensible effectiveness. Therefore, the exergy efficiency of the present system should be analyzed to gain a clear understand of its performance.

Figure 8 shows the exergy efficiency of the present system at different sensible effectiveness and ambient temperatures. Contrary to expectations, at low ambient temperatures (<30 °C), the exergy efficiency slightly increases with an increase in sensible effectiveness, though the changes are minimal. Since sensible heat lost in the membrane dehumidifier is gained by the ambient air, a lower temperature difference between the feed and permeate side reduces exergy destruction in the membrane dehumidifier. Additionally, sensible heat loss results in a smaller temperature difference between the air entering the condenser and dead state, leading to a lower refrigerant condensing temperature and a reduced temperature difference between the refrigerant at the condenser and the dead state. Although exergy input is higher at higher sensible effectiveness, the smaller temperature difference between the working fluids and the dead state leads to a comparatively lower exergy destruction and high exergy efficiency. However, at low sensible effectiveness, as the dead state temperature increases (≥30 °C), the temperature differences become minimal, making the membrane dehumidifier with lower sensible effectiveness more efficient. The highest exergy efficiency of 10.32% was achieved with a membrane sensible effectiveness of 0.1 and an ambient temperature of 35 °C.

### 3.5. Exergy Flow and Sources of Exergy Destruction

**Figure 9:**
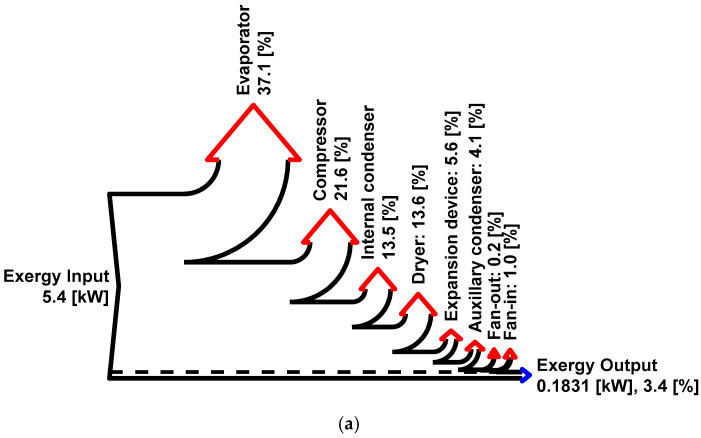

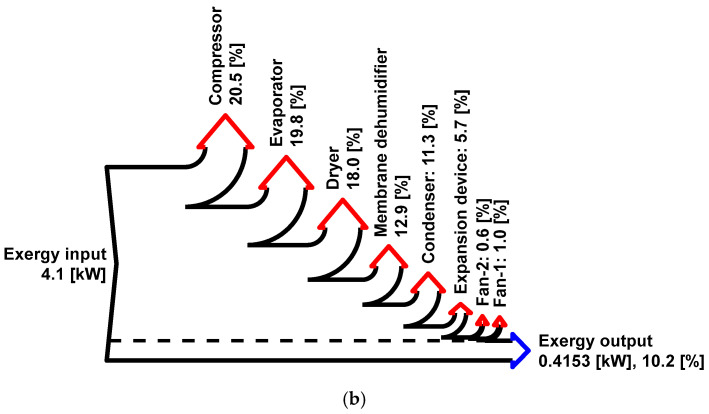
Grassman diagram of the (**a**) baseline and (**b**) present system at 35 °C ambient temperature and 5% relative humidity. The other variables are kept constant, as mentioned in Figure 3.

A detailed exergy flow of the components in the baseline and present system is represented in a Grassman diagram, which is an exergy version of the Sankey diagram (Figure 9). In the baseline system, the evaporator and other heat pump components contribute to significant exergy destruction due to the large temperature difference between the refrigerant at the evaporator (20 °C) and the dead state (35 °C). However, in the present system, the evaporator exchanges heat with ambient air, resulting in lower exergy destruction. In the present system, the temperature difference (3.2 °C) between the air approaching the evaporator and the dead state is much smaller than in the baseline system (19.2 °C). Additionally, the dehumidification process in the present system occurs at a higher temperature, resulting in 25% less exergy destruction compared to the dehumidification process in the baseline system. The reduced temperature loss using the membrane dehumidifier allows hotter air to enter the condenser, significantly reducing exergy destruction in the condenser. The exergy destruction in the dryer (0.72 kW) is the same in both systems. However, because the total exergy input is lower in the present system, the percentage of exergy destruction in the dryer appears higher than in the baseline system. The reduced exergy input and lower exergy destruction in the heat pump components contribute to higher exergy efficiency in the present system.

**Figure 10:**
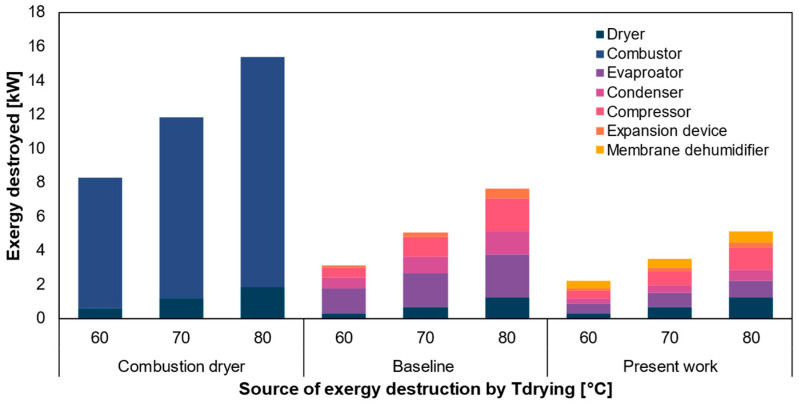
Exergy destruction in the major components of the natural gas-fired dryer, baseline heat pump dryer and the present systems at different drying temperatures (60 °C, 70 °C and 80 °C), with an ambient condition of 35 °C and 5% relative humidity. The other variables for the baseline and proposed system are kept constant, as mentioned in Figure 3.

Furthermore, the exergy destruction of the natural gas-fired dryer, baseline heat pump dryer and the present system at different drying temperatures is compared and presented in Figure 10. The natural gas dryer comprises a combustor, a heat exchanger and a drying chamber [54]. In this system, the combustor burns natural gas to supply the required heat to the heat exchanger, where ambient air is heated to the desired temperature (70 °C) before being delivered to the drying chamber. Exergy destruction is calculated assuming a calorific value of 55,448 kJ/kg for natural gas [55], an air fuel ratio of 17.2:1 in the combustor [56], a combustion efficiency of 80% [57], and a heat exchanger effectiveness of 0.8 [58]. Further details on exergy destruction calculations of the natural gas dryer are provided in the Supplementary Section. In the natural gas dryer, the high operating temperatures of the combustion chamber contribute to significant exergy destruction. This study examines an open-vented natural gas dyer, where ambient air (at 35 °C and 5% RH) is heated and supplied directly to the dryer without dehumidification. Under these ambient conditions, the air entering the dryer is exceptionally dry, with a vapor pressure 54% lower than that of the baseline and proposed systems. This condition causes rapid moisture removal but also leads to substantial exergy destruction in the drying chamber compared to the baseline and proposed system. Furthermore, as discussed before, the exergy destruction in the dryer is identical for both the baseline and proposed systems across different drying temperatures. However, at higher drying temperatures, the larger temperature difference between the ambient air and working fluid leads to increased exergy destruction. Notably, most of the exergy destruction occurs in the evaporator, compressor, and condenser, which is significantly reduced in the proposed system.

## 4. Conclusions

A second law analysis was conducted for a convective heat pump dryer coupled with a membrane dehumidifier and compared with the baseline system. This study examined irreversibility in each component to identify those contributing most to the exergy loss and to gain insights into performance. The key conclusions are as follows:

- The irreversibility in the dryer at different dead state conditions is identical for both systems.
- The inclusion of an MERV in the present system plays a crucial role, specifically transferring heat and moisture to the ambient air, which drastically reduces exergy destruction in the evaporator. Furthermore, reduced heat loss during the dehumidification process requires a smaller heat pump unit and lower exergy input.
- Low drying temperatures may contribute to less exergy destruction in each component compared to high drying temperatures (≥70 °C), which can be further enhanced with an MERV that has high sensible and latent effectiveness.
- Compared to the baseline system, the present system reduces heat loss by 35% (at 35 °C and 40% RH) during the dehumidification process. This significantly improved system performance, reducing irreversibilities by 53% in the evaporator and 33% in the compressor.
- The present system requires the lowest exergy input (3.94 kW) at the highest ambient temperature (35 °C) and relative humidity (40% RH), which is 23% lower than the baseline system under the same operating condition.
- In the present system, the evaporator’s heat exchange with the ambient air is beneficial only at an ambient temperature above 20 °C. Otherwise, at lower temperatures (<5–15 °C), the evaporator operates at a much lower temperature than the baseline system, causing higher exergy destruction and reduced exergy efficiency.
- The present system shows up to a 66.7% improvement in exergy efficiency compared to the baseline system at an ambient condition of 35 °C and 40% relative humidity.
- The present system is most effective in terms of exergy efficiency when the ambient temperature exceeds 20 °C and relative humidity is below 40%, achieving up to a 35% reduction in sensible heat loss using the membrane dehumidifier.

## Figures and Tables

**Table 1:** Energy and exergy balance of each component of the present system.

Components		Energy Balance	Exergy Balance
Dryer/evaporative cooler	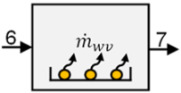	${\dot{m}}_{6}h_{6}+{\dot{m}}_{wv}h_{fg}={\dot{m}}_{7}h_{7}$	${\dot{Ex}}_{6}+{\dot{Ex}}_{evap}={\dot{Ex}}_{7}+{\dot{I}}_{dryer}$
Evaporator	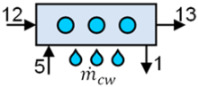	${\dot{m}}_{12}h_{12}+{\dot{m}}_{5}h_{5}={\dot{m}}_{13}h_{13}+{\dot{m}}_{1}h_{1}+{\dot{m}}_{cw}h_{cw}$	${\dot{Ex}}_{12}+{\dot{Ex}}_{5}={\dot{Ex}}_{13}+{\dot{Ex}}_{1}+{\dot{Ex}}_{cw}+{\dot{I}}_{evap}$
Condenser	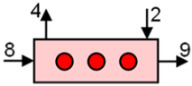	${\dot{m}}_{8}h_{8}+{\dot{m}}_{2}h_{2}={\dot{m}}_{9}h_{9}+{\dot{m}}_{4}h_{4}$	${\dot{Ex}}_{8}+{\dot{Ex}}_{2}={\dot{Ex}}_{9}+{\dot{Ex}}_{4}+{\dot{I}}_{cond}$
Compressor	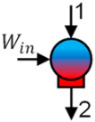	$W_{c}+{\dot{m}}_{1}h_{1}={\dot{m}}_{2}h_{2}$	$W_{c}+{\dot{Ex}}_{1}={\dot{Ex}}_{2}+{\dot{I}}_{comp}$
Expansion device	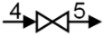	${\dot{m}}_{4}h_{4}={\dot{m}}_{5}h_{5}$	${\dot{Ex}}_{4}={\dot{Ex}}_{5}+{\dot{I}}_{exp}$
Fan-1	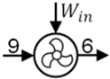	$W_{f,1}+{\dot{m}}_{9}h_{9}={\dot{m}}_{6}h_{6}$	$W_{f,1}+{\dot{Ex}}_{9}={\dot{Ex}}_{6}+{\dot{I}}_{f,1}$
Fan-2	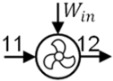	$W_{f,2}+{\dot{m}}_{11}h_{11}={\dot{m}}_{12}h_{12}$	$W_{f,2}+{\dot{Ex}}_{11}={\dot{Ex}}_{12}+{\dot{I}}_{f,2}$
Membrane dehumidifier	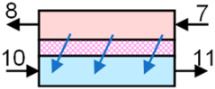	${\dot{m}}_{10}h_{10}+{\dot{m}}_{7}h_{7}={\dot{m}}_{11}h_{11}+{\dot{m}}_{8}h_{8}$	${\dot{Ex}}_{10}+{\dot{Ex}}_{7}={\dot{Ex}}_{11}+{\dot{Ex}}_{8}+{\dot{I}}_{md}$

**Table 2:** State point properties of the air and refrigerant in the proposed system shown in Figure 1.

State	Flow Rate [kg/s]	Temperature [°C]	Relative Humidity [%]	Specific Enthalpy [kJ/kg]	Specific Entropy [kJ/kg·K]	Specific Exergy [kJ/kg]
1	0.0519	18	-	436.91	1.8159	0.3289
2	0.0519	94.7	-	492.59	1.2713	2.2329
4	0.0519	64	-	283.18	1.2713	0.6307
5	0.0519	18	-	283.18	1.2880	0.3777
6	0.2512	70	10	119	0.4025	1.1116
7	0.2545	39.75	69.73	119	0.4112	0.5056
8	0.2512	27	87.70	75.76	0.2664	0.0975
9	0.2512	69.25	10.31	119.02	0.4020	0.9840
10	0.2417	20	95	54.40	0.1948	0
11	0.2450	33.62	81.01	100.10	0.3483	0.1782
12	0.2450	34.12	79.07	100.62	0.3489	0.2613
13	0.2427	22.90	100	67.61	0.2397	0.0137

## Data Availability

The supplementary data related to this work will be provided based upon request.

## Supplementary Materials

The following supporting information can be downloaded at: https://www.mdpi.com/article/10.3390/e27020197/s1, Figure S1: The schematic of the natural gas fired vented type dryer. See [55,56,57,58,59,60,61,62].

## Nomenclature

Symbols	
$h_{o}$	Specific enthalpy at dead state, [kJ/kg·K]
$\dot{Ex}$	Exergy transfer rate, [kW]
$\dot{I}$	Irreversibility rate, [kW]
*Q*	Heat transfer rate, $\left[kW\right]$
$\dot{V}$	Volumetric flow rate, $\left[m^{3}/s\right]$
*W*	Work, $\left[kW\right]$
$c_{p}$	Specific heat, $\left[kJ/kg\cdot K\right]$
$c_{p-h}$	Specific humidity capacity, $\left[{kg}_{water}/{kg}_{air}\cdot kPa\right]$
$\dot{m}$	Mass flow rate, $\left[kg/s\right]$
$P_{v}$	Vapor partial pressure, $\left[kPa\right]$
$s_{o}$	Specific entropy at dead state, [kJ/kg·K]
∆P	Pressure drop, $\left[Pa\right]$
*e*	Specific exergy, [kJ/kg]
*h*	Specific enthalpy, $\left[kJ/kg\right]$
	Mass, $\left[kg\right]$
*s*	Specific entropy, [kJ/kg·K]
*T*	Temperature, °C
t	Time, $\left[h\right]$
w.b	Wet basis
Greek symbols	
*ɛ*	Effectiveness, $\left[-\right]$
*η*	Efficiency, $\left[\%\right]$
*ω*	Humidity ratio, $\left[{kg}_{water}/{kg}_{air}\right]$
Subscripts	
a	Air
comp	Compressor
cond	Condenser
d	Drying
eq	Equivalent
evap	Evaporator
II	Second law
min	Minimum
r	Refrigerant
sat	Saturation
v	Vapor
w	Water
wv	Water vapor
Abbreviations	
COP	Coefficient of performance
ERV	Energy recovery ventilator
HPD	Heat pump dryer
HVAC	Heating, ventilation and air conditioning
MERV	Membrane energy recovery ventilator
PAN	Polyacrylonitrile
PBI	Polybenzimidazole
PVA	Polyvinyl alcohol
PVDF	Polyvinylidene fluoride
SMER	Specific moisture extraction ratio
SPEEK	Sulfonated poly ether ether ketone
T-s	Temperature–entropy
