# Supplementary material for: Exergy Analysis of a Convective Heat Pump Dryer Integrated with a Membrane Energy Recovery Ventilator
Source: Entropy (Basel). 2025 Feb 13;27(2):197. doi: 10.3390/e27020197 (PMC11854696; doi:10.3390/e27020197)
Supplement: Supplementary file 1 [file entropy-27-00197-s001.zip › entropy-3379793-supplementary.pdf]

Supplementary Materials for Exergy Analysis of a Convective Heat Pump Dryer Integrated with a Membrane Energy Recovery Ventilator

The following content describes the modeling for a gas-fired dryer, which was used as a common baseline for standard drying processes.

In Fig. 10, the sources of exergy destruction of the proposed system are compared with the baseline heat pump dryer and a natural gas fired dryer. Exergy destruction in the major components of the natural gas fired dryer, baseline heat pump dryer and the present systems at different drying temperatures (60℃, 70℃ and 80℃), with an ambient condition of 35℃ and 5% relative humidity are compared. The natural gas dryer comprises a combustor, a heat exchanger and a drying chamber. The combustor burns natural gas to supply the required heat to the heat exchanger, where ambient air (here, 35℃ and 5% RH) is heated to the desired temperature (70℃) before being delivered to the drying chamber. A schematic of the natural gas fired dryer is represented in Fig.S1.

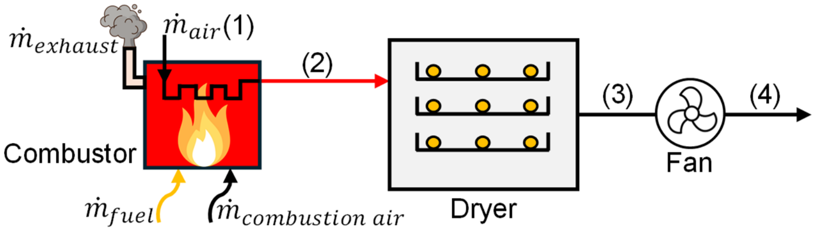

Figure S1. The schematic of the natural gas fired vented type dryer.

The following details are assumed to calculate the exergy destruction in the natural gas fired dryer.

- Higher heating value (HHV) of natural gas: 52,210 kJ/kg[1].
- Air fuel ratio in the combustor: 17.2:1 [2].
- Combustion efficiency: 80%[3]
- Heat exchanger effectiveness: 0.8[4].
- The dryer air inlet temperature (T_2) varied at 60℃, 70℃ and 80℃ respectively.
- Combustion temperature: 1800 K[5]
- Exhaust gas temperature: 473 K[6].
- Dead state temperature, relative humidity and pressure are considered to be equal to the ambient conditions.

Initially, the total heat required ($\dot{Q}_{\text{required}}$) to be supplied by the combustor is calculated as

$$\dot{Q}_{\text{required}} = \dot{m}_{\text{air}} c_{\text{p-air}} (T_2 - T_1) \tag{S1}$$

Here, $\dot{m}_{air}$ is the air mass flow rate, c_{p-air} is the specific heat of air, T is the air temperature, respectively. Then, the total fuel required to supply the calculated heat load is determined using the combustion efficiency and calorific value of the fuel. The required fuel flow rate and combustion air flow rate are calculated as follows.

$$\dot{m}_{\text{fuel}} = \frac{\dot{Q}_{\text{required}}}{\eta_{\text{combustion}}} \tag{S2}$$

$$\dot{m}_{\text{combustion air}} = \dot{m}_{\text{fuel}} \times air\ fuel\ ratio \tag{S3}$$

The total exhaust flow rate is

$$\dot{m}_{\text{exhaust}} = \dot{m}_{\text{fuel}} + \dot{m}_{\text{combustion air}} \tag{S4}$$

Next, the specific exergy at each states in the natural gas dryer is calculated to estimate the exergy destruction in each components. The specific air exergy is calculated as described in the methodology section of the proposed membrane-based heat pump dryer. Additionally, the specific exergy of the fuel is calculated by assuming only the chemical exergy[7].

$$\dot{Ex}_{\text{fuel}} = \dot{m}_{\text{fuel}} HHV \left(1 - \left(\frac{T_{\text{o}}}{T_{\text{combustion}}}\right)\right) \tag{S5}$$

Here, $\dot{Ex}$ is specific exergy. The specific exergy of the exhaust gas stream is calculated by considering only its thermal exergy, neglecting its chemical potential. This assumes the flue gas can be used solely for heat recovery applications[8].

$$\dot{Ex}_{\text{exhaust}} = \dot{m}_{\text{exhaust}} c_{\text{p-exhaust}} (T_{\text{exhaust}} - T_{\text{o}}) \left(1 - \left(\frac{T_{\text{o}}}{T_{\text{exhaust}}}\right)\right) \tag{S6}$$

The exergy balance of the combustor, dryer and fan are described as

$$\dot{Ex}_{\text{fuel}} + \dot{Ex}_{\text{combustion air}} + \dot{Ex}_{\text{air,1}} = \dot{Ex}_{\text{exhaust gas}} + \dot{Ex}_{\text{air,2}} + \dot{I}_{\text{combustor}} \tag{S7}$$

$$\dot{Ex}_{\text{evap}} + \dot{Ex}_{\text{air,2}} = \dot{Ex}_{\text{air,3}} + \dot{I}_{\text{dryer}} \tag{S8}$$

$$W_{\text{fan}} + \dot{Ex}_{\text{air,3}} = \dot{Ex}_{\text{air,4}} + \dot{I}_{\text{fan}} \tag{S9}$$

Here, $\dot{I}$ is exergy destruction, and W is work, respectively. The total exergy destruction is calculated as

$$\dot{I}_{\text{total}} = \dot{I}_{\text{combustor}} + \dot{I}_{\text{dryer}} + \dot{I}_{\text{fan}} \tag{S10}$$

References:

[1] Alhajeri NS, Dannoun M, Alrashed A, Aly AZ. Environmental and economic impacts of increased utilization of natural gas in the electric power generation sector: Evaluating the benefits and trade-offs of fuel switching. J Nat Gas Sci Eng 2019;71. https://doi.org/10.1016/j.jngse.2019.102969.

[2] Hoda A, Rahman TMR, Asrar W, Khan SA. A Comparative Study of Natural Gas and Biogas Combustion in A Swirling Flow Gas Turbine Combustor. Combustion Science and Technology 2022;194:2613–40. https://doi.org/10.1080/00102202.2021.1882441.

[3] Brand L, Rose W. Measure Guideline: High Efficiency Natural Gas Furnaces Partnership for Advanced Residential Retrofit. 2012.

[4] Borodulin VY, Nizovtsev MI. A criterial analysis of the effectiveness of air-to-air heat exchangers with periodic change of airflow direction. Appl Therm Eng 2018;130:1246–55. https://doi.org/10.1016/j.applthermaleng.2017.11.126.

[5] Weber R, Orsino S, Lallemant N, Verlaan AD. Combustion of natural gas with high-temperature air and large quantities of flue gas. vol. 28. 2000. https://doi.org/https://doi.org/10.1016/S0082-0784(00)80345-8.

[6] Che D, Liu Y, Gao C. Evaluation of retrofitting a conventional natural gas fired boiler into a condensing boiler. Energy Convers Manag 2004;45:3251–66. https://doi.org/10.1016/j.enconman.2004.01.004.

[7] Dincer I, Hussain MM, Al-Zaharnah I. Energy and exergy utilization in agricultural sector of Saudi Arabia. Energy Policy 2005;33:1461–7. https://doi.org/10.1016/j.enpol.2004.01.004.

[8] El Fil B, Garimella S. Energy-efficient gas-fired tumble dryer with adsorption thermal storage. Energy 2022;239. https://doi.org/10.1016/j.energy.2021.121708.
